# The meningeal lymphatic vessel–peripheral immune axis: a novel therapeutic target in neurodegenerative diseases

**DOI:** 10.1186/s12974-026-03849-5

**Published:** 2026-05-04

**Authors:** Zhidong He, Jing Sun

**Affiliations:** 1 Department of Neurosurgery, China-Japan Union Hospital of Jilin University, No. 126 Xiantai Street, Changchun, 130031 Jilin China; 2 Department of Neurology, China-Japan Union Hospital of Jilin University, No. 126 Xiantai Street, Changchun, 130031 Jilin China

**Keywords:** Meningeal lymphatic vessels, Peripheral immunity, Neuroinflammation, Neurodegenerative diseases, VEGF-C, Therapeutic target, Experimental autoimmune encephalomyelitis, Multiple sclerosis

## Abstract

Meningeal lymphatic vessels serve as a direct anatomical conduit connecting the central nervous system and the peripheral immune system, fundamentally challenging the traditional view of the brain as an "immune-privileged" organ. This review systematically examines, for the first time, the meningeal lymphatic vessel–peripheral immune axis as an integrated framework linking central proteinopathy, neuroinflammation, and systemic immune responses in neurodegenerative diseases. We highlight recent therapeutic advances, including lymphatic regeneration via the VEGF-C pathway, peripheral immune modulation, and combinatorial approaches. We also discuss current challenges and future translational directions, emphasizing the need for integrating lymphatic imaging with immune phenotyping to enable personalized interventions. While the majority of evidence discussed derives from preclinical models, we critically evaluate its translational relevance and highlight unresolved controversies. Based on the evidence, we propose that targeting this axis offers a dual opportunity to enhance CNS waste clearance and restore immune tolerance, providing a promising framework for clinical management.

## Introduction

Neurodegenerative diseases are a class of disorders characterized by the progressive loss of specific neuronal populations, including Alzheimer’s disease, Parkinson's disease, Huntington's disease, and amyotrophic lateral sclerosis. For a long time, research into the pathological mechanisms of these diseases has primarily focused on intrinsic molecular events within neurons, such as the aggregation and spread of abnormal proteins, energy metabolism crises caused by mitochondrial dysfunction, oxidative stress damage, and impaired axonal transport [1]. However, with deeper investigation into disease mechanisms, neuroinflammation has been confirmed as one of the core common pathological features of various neurodegenerative diseases [2–6]. Chronic activation of microglia, reactive proliferation of astrocytes, and the release of a series of inflammatory cytokines not only directly damage neurons but also accelerate disease progression through positive feedback loops [7–10]. In this context, a fundamental question emerges: How exactly do inflammatory signals within the brain, traditionally considered an immune-privileged organ, connect with the body's overall immune status? Does the peripheral immune system participate in the maintenance and amplification of central nervous system (CNS) inflammation?

In 2015, research teams led by Louveau at the University of Virginia and Aspelund at the University of Helsinki independently published landmark findings, confirming the existence of functional lymphatic vessels within the dura mater of mammals [11, 12]. This discovery fundamentally revised the anatomical understanding of the CNS, as the academic community previously widely believed the brain lacked classical lymphatic vessels. Notably, the foundational insights into meningeal lymphatic function and perineural drainage pathways were initially derived from experimental autoimmune encephalomyelitis (EAE) models of multiple sclerosis, underscoring the close relationship between neuroinflammation and lymphatic biology [13, 14]. These newly identified meningeal lymphatic vessels primarily run along the venous sinuses, collecting metabolic waste, soluble proteins, and immune cells from the cerebrospinal fluid and draining them to the deep cervical lymph nodes [15–18]. More importantly, the discovery of this structure not only provided a definitive anatomical basis for the existence of a brain waste clearance system but, crucially, revealed a direct channel for material exchange and information communication between the CNS and the peripheral immune system. Since then, research on meningeal lymphatic vessels has rapidly become a frontier hotspot in neuroscience, and their role in the pathogenesis and progression of neurodegenerative diseases has gained increasing attention [19–23].

In recent years, the association between meningeal lymphatic dysfunction and various neurodegenerative diseases—including Alzheimer’s disease, Parkinson's disease, and amyotrophic lateral sclerosis—has been progressively elucidated [24]. Studies demonstrate that during aging, the structural integrity of meningeal lymphatic vessels begins to decline, characterized by decreased lymphatic vessel density, luminal collapse, and reduced drainage efficiency [16, 23, 25]. Notably, meningeal lymphatic vessels are not merely passive drainage channels but rather active regulatory platforms involved in meningeal lymphatic–peripheral immune axis [18, 26]. This understanding has given rise to the concept of the “meningeal lymphatic vessel–peripheral immune axis”, which integrates focal pathological changes in the CNS with systemic peripheral immune responses into a unified pathophysiological framework.

Unlike previous reviews that focused separately on glymphatic function or neuroinflammation, we herein synthesize emerging evidence to propose a bidirectional vicious cycle wherein lymphatic dysfunction exacerbates both central pathology and peripheral immune dysregulation, and conversely, peripheral immune activation further compromises lymphatic integrity. We argue that restoring lymphatic function combined with immunomodulation may break this cycle and offer a dual therapeutic opportunity. While the majority of evidence discussed derives from preclinical models, we critically evaluate its translational relevance and highlight unresolved controversies. Given that the most robust evidence currently exists for Alzheimer's disease, this review will focus primarily on AD while noting parallel findings in Parkinson's disease and amyotrophic lateral sclerosis where available. While the glymphatic system is an integral part of CNS waste clearance, this review will focus specifically on meningeal lymphatic vessels and their interactions with peripheral immunity. The glymphatic system is introduced only as a functional partner; readers seeking a comprehensive review are referred elsewhere (e.g., [27, 28]).

### Search strategy and selection criteria

This is a narrative review informed by a systematic literature search. We aimed to provide a comprehensive overview rather than a quantitative meta-analysis. We searched PubMed, Web of Science, and Scopus databases for articles published between January 2015 and February 2026, using combinations of the following keywords: “meningeal lymphatic vessels”, “glymphatic system”, “neurodegenerative diseases”, “Alzheimer’s disease”, “Parkinson's disease”, “Experimental autoimmune encephalomyelitis”, “Multiple sclerosis”, “neuroinflammation”, “peripheral immune cells”, and “VEGF-C”, and “immune tolerance”. The search was limited to original research articles and reviews published in English.

#### Inclusion and exclusion criteria

Studies were included if they: (1) investigated meningeal lymphatic structure or function in the context of neurodegenerative diseases or aging; (2) examined the relationship between meningeal lymphatic vessels and peripheral immune responses; (3) evaluated therapeutic interventions targeting meningeal lymphatic vessels or associated immune pathways; or (4) provided mechanistic insights into meningeal lymphatic biology relevant to neurodegeneration. Studies were excluded if they: (1) were conference abstracts, case reports, or opinion pieces without original data; (2) focused exclusively on peripheral lymphatic vessels without meningeal involvement; or (3) lacked direct relevance to neurodegenerative disease mechanisms. The selection was performed by two authors, and discrepancies were resolved through discussion.

## Anatomical and physiological basis of meningeal lymphatic vessels and their immune regulatory functions

### Anatomical structure and distribution characteristics

Meningeal lymphatic vessels are located within the inner layer of the dura mater, forming an intricate lymphatic network that covers the cerebral convexity and skull base [15, 29]. Specifically, these lymphatic vessels predominantly course along the venous sinuses—including the superior sagittal sinus, transverse sinus, and sigmoid sinus—establishing drainage pathways that parallel the venous system. In the skull base region, they are also distributed around the olfactory bulb, adjacent to the cribriform plate, and at sites where cranial nerves exit the skull. Histologically, meningeal lymphatic vessels consist of a single layer of lymphatic endothelial cells that specifically express lymphatic endothelial markers including LYVE-1, PROX1, PDPN, and VEGFR-3 [11, 12, 15, 30, 31]. Compared to lymphatic vessels in peripheral tissues such as skin and intestine, meningeal lymphatic vessels have relatively smaller diameters, typically ranging from 50—100 µm, and their density is markedly lower than that of peripheral lymphoid tissues. Additionally, meningeal lymphatic vessels contain valve structures—unidirectional flaps formed by endothelial cell folds—that ensure unidirectional lymph flow and prevent backflow.

A direct anatomical communication exists between meningeal lymphatic vessels and the subarachnoid space. Tracer injection experiments confirm that tracers administered into the cerebrospinal fluid rapidly enter meningeal lymphatic vessels and reach the deep cervical lymph nodes within hours [32]. Several mechanisms may underlie this communication: First, the sheath spaces of olfactory nerves traversing the cribriform plate represent important routes for cerebrospinal fluid egress [33, 34]. Second, similar spaces exist at the exit sites of certain cranial nerves, including the trigeminal, facial, glossopharyngeal, and vagus nerves [35, 36]. Third, recent studies suggest that arachnoid granulations—long recognized as sites of CSF absorption into the venous system—may also serve as lymphatic conduits communicating with the dura-arachnoid stroma and bone marrow [37, 38]. Furthermore, emerging evidence indicates that CSF exits the subarachnoid space into meningeal lymphatic vessels via specialized arachnoid cuff exit points surrounding cerebral veins, though this concept has not yet been universally accepted [38]. Notably, there is significant spatial heterogeneity in lymphatic drainage efficiency across different brain regions [39, 40]. Studies utilizing dynamic contrast‑enhanced MRI reveal that regions such as the basal forebrain, hippocampus, and entorhinal cortex—precisely the areas where Alzheimer’s disease pathology first emerges—exhibit relatively lower lymphatic drainage efficiency [41]. This anatomical feature offers a novel perspective for understanding the mechanisms underlying selective regional vulnerability in neurodegenerative diseases, suggesting that inherent differences in clearance capacity may be a crucial factor determining the spatial distribution pattern of pathological proteins.

**Fig. 1:**
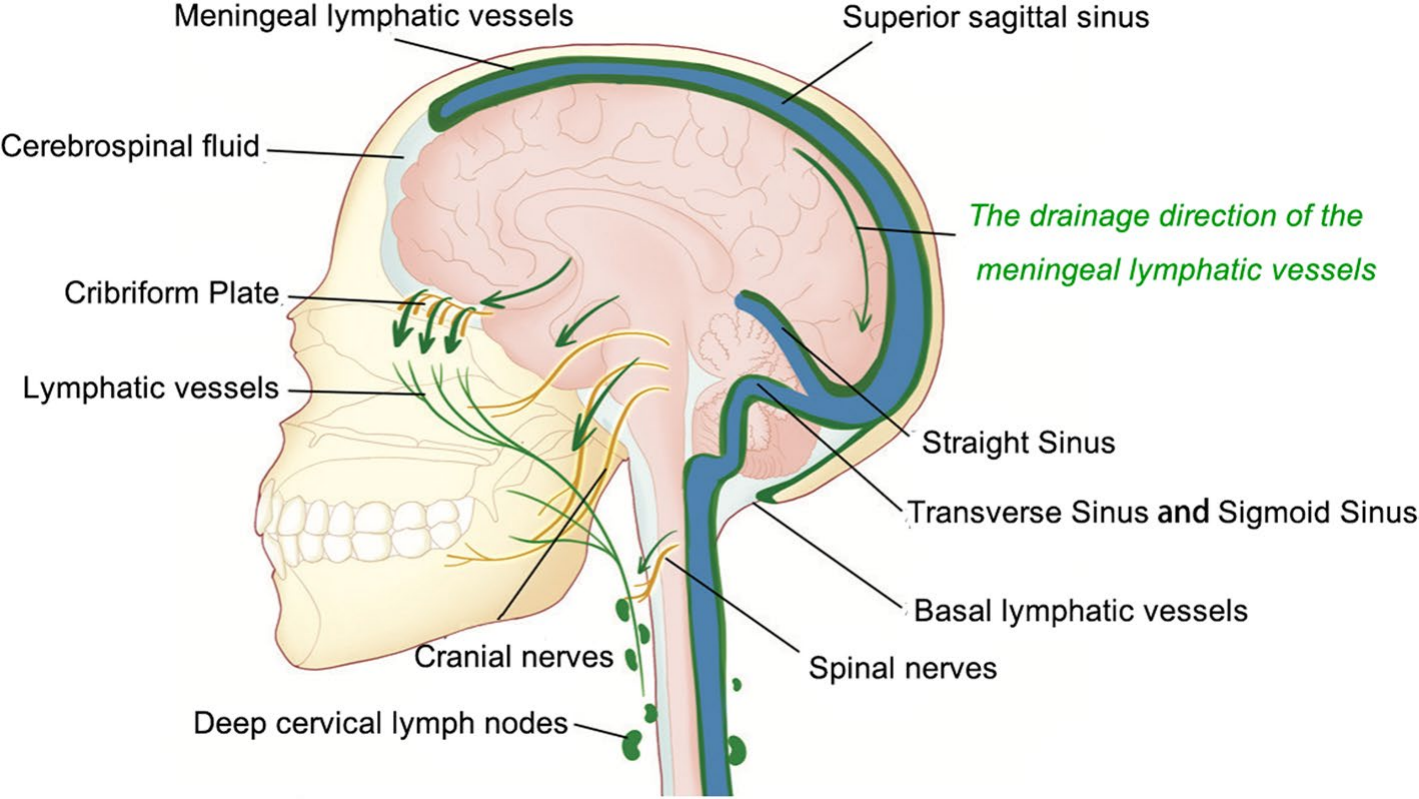
Anatomical distribution of meningeal lymphatic vessels. Schematic coronal view of the skull showing meningeal lymphatic vessels (green, LYVE‑1⁺/PROX1⁺) coursing along the dural venous sinuses, including the superior sagittal sinus, straight sinus, transverse sinus, and sigmoid sinus. Basal lymphatic vessels located near the skull base drain cerebrospinal fluid via the cribriform plate and along cranial nerve sheaths. Cerebrospinal fluid and interstitial fluid are transported through these vessels to the deep cervical lymph nodes. Arrows indicate the direction of lymphatic drainage

A schematic illustration of the anatomical distribution of meningeal lymphatic vessels is provided in Fig. 1. This figure depicts the location of LYVE‑1⁺/PROX1⁺ vessels along the superior sagittal sinus, transverse sinus, and sigmoid sinus, as well as the olfactory bulb and cribriform plate region. Key routes of cerebrospinal fluid egress, including olfactory nerve sheath spaces and arachnoid cuff exit points, are also shown. The inset highlights the relationship between meningeal lymphatics and deep cervical lymph nodes.

### Lymphatic drainage and waste clearance function

The core function of meningeal lymphatic vessels is to facilitate efflux of metabolic products from brain interstitial fluid. This process operates in coordination with the glymphatic system, which directs CSF influx along peri‑arterial spaces and exchanges with interstitial fluid via astrocytic aquaporin‑4 [27, 42, 43]. Subsequently, interstitial fluid carrying Aβ, Tau, and α‑synuclein drains into meningeal lymphatics and is transported to deep cervical lymph nodes [19, 44–46].

### Immune surveillance and central-peripheral crosstalk

Beyond serving as passive waste clearance channels, meningeal lymphatic vessels represent a dynamic platform actively involved in immune regulation. Recent researchers consider meningeal lymphatics as a communicator between the brain and peripheral immunity [47, 48]. Under physiological conditions, small amounts of CNS antigens continuously travel via meningeal lymphatic vessels to the deep cervical lymph nodes. These CNS antigens include fragments of myelin proteins, soluble components released by neurons, and antigen fragments processed by microglia. Upon arrival at the lymph nodes, these antigens are captured and processed by dendritic cells within the nodes and presented to naïve T cells in the form of MHC molecule complexes [28, 49]. This process likely contributes to maintaining immune tolerance towards CNS-specific antigens. Studies reveal that within the deep cervical lymph nodes of normal mice, there is a considerable number of regulatory T cells (Tregs) recognizing myelin oligodendrocyte glycoprotein, myelin basic protein, and neuronal antigens [50]. These Foxp3-positive Tregs, by secreting inhibitory cytokines like IL-10 and TGF-β, maintain a state of immune unresponsiveness to CNS antigens, preventing autoimmune reactions [51]. This Treg-mediated tolerance is a key mechanism that prevents spontaneous neuroinflammation under physiological conditions.

**Fig. 2:**
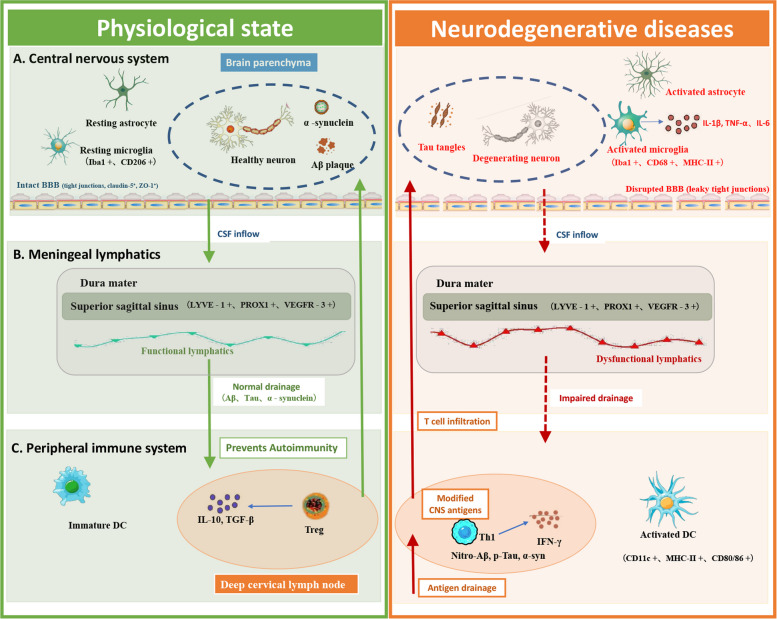
Meningeal lymphatic vessel-peripheral immune axis mechanism. **A** Central nervous system. In physiological conditions (left), healthy neurons (NeuN⁺), resting microglia (Iba1⁺, CD206⁺, ramified morphology), and an intact blood–brain barrier (tight junctions, claudin‑5⁺, ZO‑1⁺) maintain immune homeostasis. In neurodegenerative diseases (right), degenerating neurons accumulate pathological proteins (Aβ plaques, hyperphosphorylated Tau tangles, α‑synuclein aggregates). Activated microglia (Iba1⁺, CD68⁺, MHC‑II⁺, amoeboid morphology) and reactive astrocytes (GFAP⁺) release pro‑inflammatory cytokines (IL‑1β, TNF‑α, IL‑6), establishing a neuroinflammatory microenvironment. **B** Meningeal lymphatics. Functional meningeal lymphatic vessels (LYVE‑1⁺, PROX1⁺, VEGFR‑3⁺) that course along the superior sagittal sinus normally mediate efficient drainage of CNS‑derived waste (Aβ, Tau, α‑synuclein) and antigens to deep cervical lymph nodes (dCLNs). In disease states (right), dysfunctional vessels exhibit structural damage, luminal collapse, reduced expression of lymphatic markers, and impaired drainage capacity (indicated by dashed lines). Impaired flow leads to the accumulation of antigens within the perivascular space and delayed transport to dCLNs. **C** Peripheral immune system. Under pathological conditions, abnormally modified CNS antigens (e.g., nitrated Aβ, p‑Tau, oligomeric α‑synuclein) drain via meningeal lymphatics to dCLNs. These modified antigens are captured by activated dendritic cells (CD11c⁺, MHC‑II⁺, CD80/86⁺), which process and present them to naïve T cells via MHC molecules. This promotes differentiation into Th1 (IFN‑γ⁺, T‑bet⁺) and Th17 (IL‑17⁺, RORγt⁺) effector cells. Activated effector T cells upregulate chemokine receptors (CXCR3, CCR5) and adhesion molecules (α4β1 integrin), enabling their migration across the blood–brain barrier into the CNS. Once in the CNS, they encounter cognate antigens presented by microglia or infiltrating dendritic cells, leading to reactivation and release of IFN‑γ and IL‑17, which perpetuate neuroinflammation and neuronal damage. Arrows indicate direction of cell trafficking and signaling

When pathological changes occur in the brain, this CNS-peripheral immune crosstalk pattern changes significantly. In neurodegenerative diseases, damaged or apoptotic neurons release large quantities of abnormally modified self-antigens, and microglial activation further alters antigen processing and presentation [52–54]. These pathologically generated CNS antigens are not only increased in quantity but, more critically, their molecular modification status is altered, such as nitration, glycosylation, or phosphorylation of Aβ, hyperphosphorylation of Tau, and conformational changes in α-synuclein. When these abnormally modified antigens reach the deep cervical lymph nodes via meningeal lymphatic vessels, they are more readily recognized by the immune system as “danger signals” rather than “self-components”. Concurrently, lymphatic endothelial cells, stimulated by inflammatory factors, upregulate adhesion molecules ICAM-1, VCAM-1, and chemokine CCL21, promoting the migration and maturation of antigen-presenting cells like dendritic cells. This process amplifies local CNS pathological signals into a systemic immune response, constituting the core of "CNS-peripheral" immune crosstalk [55]. Thus, meningeal lymphatic vessels are not merely physical conduits connecting the CNS and periphery but are critical platforms regulating the balance between immune tolerance and immune activation. The interplay between CNS antigens, meningeal lymphatics, and peripheral immune cells is schematically depicted in Fig. 2.

## Pathological mechanisms of meningeal lymphatic dysfunction in neurodegenerative diseases

### Structural damage and drainage failure of lymphatic vessels

In various neurodegenerative diseases, structural remodeling and functional impairment of meningeal lymphatic vessels are consistently observed. Evidence from human autopsy studies shows that the density of LYVE-1 positive lymphatic vessels in the parasagittal region of the superior sagittal sinus is reduced in Alzheimer's disease patients compared to age-matched cognitively normal controls [56]. More notably, the remaining lymphatic vessels commonly exhibit degenerative changes, manifested as irregular luminal collapse, loose endothelial cell junctions, thickened basement membrane, and increased perivascular collagen deposition [57, 58]. In severe cases, lymphatic vessels may even show segmental occlusion, forming bead-like or saccular dilations. Electron microscopy further reveals cytoplasmic vacuolization in lymphatic endothelial cells under pathological conditions, swollen mitochondria, and abnormally widened intercellular spaces [24]. These structural damages directly lead to impaired lymphatic barrier function and increased permeability, potentially allowing untreated cerebrospinal fluid to leak directly into surrounding tissues.

In addition, in vivo functional assessments confirm significant impairment of meningeal lymphatic drainage. Using dynamic contrast-enhanced MRI, researchers inject tracers like gadopentetate dimeglumine into the subarachnoid space and track their transport along lymphatic vessels to the deep cervical lymph nodes through sequential scanning [59, 60]. In Alzheimer's disease patients, the time for tracers to reach the deep cervical lymph nodes from the parasagittal lymphatic vessels is prolonged by more than twofold, and the peak signal intensity decreases by approximately 60%, indicating a significant reduction in lymphatic drainage efficiency [56, 57]. Similar functional abnormalities are observed in Parkinson's disease patients, with the degree of drainage impairment positively correlating with disease duration and motor symptom severity [61]. Interestingly, in Huntington's disease, even before overt clinical symptoms appear, some individuals already exhibit reduced lymphatic drainage function, suggesting that lymphatic dysfunction may occur in the preclinical stage of the disease [62].

The mechanisms leading to meningeal lymphatic injury involve multiple levels and may differ across diseases. First, although direct evidence in lymphoendothelial cells is still lacking, studies in cerebrovascular endothelial cells have clearly confirmed that Aβ induces apoptosis through the mitochondrial pathway (Bax/Bcl-2/caspase-3), suggesting that lymphoendothelial cells may have similar susceptibility [63]. Second, cytokines released during chronic neuroinflammation cause injury to the lymphatic endothelium. Pro-inflammatory factors like TNF-α and IL-1β can activate the NF-κB signaling pathway in endothelial cells, upregulate matrix metalloproteinase expression, degrade the extracellular matrix, and simultaneously disrupt intercellular tight junctions [20, 64–66]. Third, age-related systemic changes also contribute [25, 35]. During aging, weakened vascular pulsations reduce the “pumping power” for lymphatic drainage; decreased cerebrospinal fluid production rates lower the load on lymphatic vessels; lymphatic endothelial cells themselves undergo senescence-related changes, such as telomere shortening and mitochondrial dysfunction. The cumulative effect of these factors makes meningeal lymphatic vessels vulnerable targets in neurodegenerative diseases.

However, several important questions and controversies remain unresolved. First, whether meningeal lymphatic dysfunction represents a primary cause or a secondary consequence of neurodegenerative pathology is still debated [56, 57, 67]. While animal studies suggest that inducing lymphatic dysfunction can accelerate Aβ deposition, it remains unclear whether this represents the initial event in human disease or occurs as part of a broader pathological cascade. Notably, some studies have failed to detect significant lymphatic structural changes in certain patient cohorts, suggesting that lymphatic involvement may be variable and context‑dependent [68]. For instance, [68] reported no significant reduction in dural lymphatic vessel density in Alzheimer's disease patients compared to controls, contrasting with the findings of Da Mesquita et al. who observed marked degeneration [56, 57]. This discrepancy may stem from differences in the anatomical regions examined (parasagittal vs. skull base), the use of post‑mortem tissue with varying autolysis times, or patient heterogeneity in terms of disease severity, genetic background (e.g., APOE4 status), and comorbidities. These conflicting results underscore the need for standardized histological and imaging protocols, as well as larger multicenter studies with well‑characterized cohorts.

Second, significant heterogeneity exists across different neurodegenerative diseases and even among patients with the same diagnosis, indicating that lymphatic involvement may vary substantially based on genetic background, age, and comorbidities [61]. Third, the temporal dynamics of lymphatic degeneration—whether it progresses linearly or in distinct phases—remain poorly characterized, and longitudinal human data are lacking. These unresolved issues highlight the need for standardized assessment methods and prospective longitudinal studies to definitively establish the causal role of meningeal lymphatic dysfunction in disease pathogenesis.

Additionally, the mechanisms driving lymphatic flow warrant further clarification. While arterial pulsations provide the primary propulsive force, it remains unclear whether meningeal lymphatic endothelial cells (LECs) are ensheathed by α‑smooth muscle actin‑expressing cells capable of autonomous contraction, or whether they rely entirely on extrinsic pulsatility. Heterogeneity in LEC coverage along different segments of the dural sinuses may contribute to regional variations in drainage efficiency, an area that requires systematic investigation.

### Aberrant central antigen presentation and breakdown of peripheral immune tolerance

The direct consequence of impaired lymphatic drainage is the abnormal retention of CNS‑derived antigenic materials within the brain and lymphatic vessels. Under normal conditions, soluble proteins generated in the brain are cleared through continuous drainage, preventing accumulation in peripheral lymphoid organs. However, when lymphatic dysfunction occurs, this clearance pathway is obstructed, leading to gradual accumulation of CNS antigens in the brain interstitial space, perivascular spaces, and within the lymphatic vessels themselves. During this retention, antigens undergo various post‑translational modifications—nitration, glycation, phosphorylation—that generate neo‑epitopes with enhanced immunogenicity. When these abnormally modified antigens eventually reach the deep cervical lymph nodes, they are recognized by dendritic cells as danger signals. Dendritic cells upregulate MHC‑II and co‑stimulatory molecules (CD80, CD86), favoring the activation of effector T cells rather than tolerance induction. Single‑cell RNA sequencing studies reveal that under neurodegenerative conditions, dendritic cells also upregulate inhibitory molecules such as PD‑L1, reflecting an attempt to counterbalance excessive inflammation, but the net effect favors Th1 and Th17 differentiation [69, 70]. Thus, the lymph node microenvironment, which should maintain immune tolerance, transforms into a site initiating and amplifying anti-CNS immune responses [48, 71, 72].

Such clonal expansion is indicative of a break in peripheral immune tolerance and provides a mechanistic link between lymphatic dysfunction and systemic autoimmunity. Once these activated antigen-specific T cells enter the bloodstream, they gain the ability to migrate directionally towards the CNS. In terms of chemokine receptor expression profiles, they upregulate receptors associated with CNS homing, such as CXCR3 and CCR5, laying the foundation for their subsequent infiltration into the brain [73, 74]. Notably, this process differs significantly from classical autoimmune diseases like multiple sclerosis: the autoimmune response targets normal myelin antigens in multiple sclerosis, while the triggering of the immune response depends on the abnormal modification and presentation of antigens under pathological conditions in neurodegenerative diseases [75, 76]. This conceptual distinction is important for treatment strategy development, as it suggests the therapeutic goal should not be complete immunosuppression, but rather the restoration of immune tolerance towards CNS antigens.

Within the deep cervical lymph nodes, antigen‑presenting cells, particularly dendritic cells (DCs), capture these modified antigens and process them for presentation via MHC class II molecules to CD4⁺ T cells. In the context of inflammation, DCs upregulate co‑stimulatory molecules such as CD80 and CD86, which interact with CD28 on naïve T cells to promote their differentiation into Th1 (IFN‑γ⁺, T‑bet⁺) and Th17 (IL‑17⁺, RORγt⁺) effector subsets. This process is further amplified by the release of pro‑inflammatory cytokines (IL‑12, IL‑23) from activated DCs, skewing the immune response toward a neuroinflammatory phenotype. In addition, cross‑presentation of CNS‑derived antigens via MHC class I may activate CD8⁺ cytotoxic T cells, contributing to neuronal damage.

### Lymphatic dysfunction as a driver of neuroinflammation: a vicious cycle

Meningeal lymphatic dysfunction serves as a primary driver of neuroinflammation by initiating a cascade of events that amplify both central and peripheral immune responses. The pathological processes described above do not operate in isolation but engage in a self‑amplifying vicious cycle. Meningeal lymphatic dysfunction leads to the accumulation and abnormal modification of CNS antigens, which in turn drive the breakdown of peripheral immune tolerance and the activation of autoreactive T cells. These activated T cells infiltrate the CNS, where they encounter their cognate antigens presented by activated microglia and dendritic cells. Upon reactivation, they release pro‑inflammatory cytokines such as IFN‑γ and TNF‑α, which further activate microglia and astrocytes, exacerbating neuroinflammation and neuronal damage. Damaged or dying neurons release additional antigens, including newly modified pathological proteins, which are drained to the peripheral lymph nodes, perpetuating the cycle. This integrated framework positions the meningeal lymphatic vessel‑peripheral immune axis as a central driver of disease progression, wherein structural damage at the lymphatic level initiates and sustains both central and peripheral immune dysregulation.

## Therapeutic strategies targeting the meningeal lymphatic vessel-peripheral immune axis

### Promoting lymphatic regeneration and repair: targeted intervention of the VEGF-C pathway

Vascular endothelial growth factor C (VEGF-C) is the master regulator of lymphangiogenesis, activating lymphatic endothelial cell proliferation, migration, and survival through specific binding to its receptor VEGFR-3. Following proteolytic cleavage, mature VEGF-C exhibits enhanced affinity for VEGFR-3, which is predominantly expressed on lymphatic endothelial cells with minimal expression on vascular endothelium—providing a molecular basis for selectively targeting lymphatic vessels [77, 78]. Based on this mechanism, VEGF-C has emerged as the preferred target for restoring meningeal lymphatic function.

Preclinical studies of exogenous VEGF-C delivery have demonstrated encouraging efficacy. In APP/PS1 transgenic Alzheimer's disease mice, stereotaxic injection of rhVEGF-C into the cisterna magna achieves sustained meningeal expression. After 1 week, immunofluorescence reveals increase in LYVE-1-positive lymphatic vessel density along in deep cervical lymph nodes, with enlarged luminal diameters and improved structural integrity [79]. Concomitantly, Here, a decline in the concentration of Aβ oligomers was detected in the brains of rhVEGF-C treated mice by ELISA. Behavioral testing demonstrates significant improvement in Morris water maze performance. Research has found that Cu2-xSe nanoparticles promote the structural and functional recovery of meningeal lymphatic vessels by stimulating the expression of VEGF-C and its receptor VEGFR3, significantly alleviating the symptoms of pre-formed fibrils (PFFs)-induced Parkinson’s disease in mice [80].

Clinical translation perspective: VEGF-C-based therapies hold promise for restoring meningeal lymphatic function, but several hurdles must be addressed before clinical application. The delivery method requires optimization to ensure safety and efficacy in humans. Options under investigation include intracisternal injection, viral vectors (e.g., AAV‑VEGF‑C), and focused ultrasound‑mediated blood–brain barrier opening to enhance gene delivery. Notably, a recent study demonstrated that chronic VEGF‑C overexpression in mice led to sustained meningeal lymphatic expansion [81]. However, this sustained manipulation of dural lymphatic vessels did not significantly alter overall brain Aβ plaque load in AD mouse models, suggesting the existence of compensatory clearance mechanisms. From a clinical translation perspective, long-term safety concerns include the potential risk of promoting tumor growth or metastasis due to systemic VEGFR‑3 activation, as well as off‑target effects on peripheral lymphatics. Patient selection may rely on imaging biomarkers of lymphatic dysfunction (e.g., DCE‑MRI) and CSF levels of Aβ/p‑tau to identify those most likely to benefit. Early‑phase clinical trials should incorporate rigorous monitoring for adverse events and employ a stepwise dose‑escalation design.

To date, the authors have not conducted their own clinical trial of VEGF‑C therapy. However, published phase I data in lymphedema (NCT04645277) suggest acceptable short‑term safety, with no dose‑limiting toxicities and a well‑tolerated profile during 12‑month follow‑up [82, 83]. To date, the authors have not conducted their own clinical trial of VEGF‑C therapy. However, published phase I data in lymphedema (NCT04645277) suggest acceptable short‑term safety, with no dose‑limiting toxicities and a well‑tolerated profile during 12‑month follow‑up [82, 83]. Regarding the ongoing AD gene therapy trial (NCT05843266), the safety of AAV‑based gene delivery for CNS diseases has been established in prior studies [84, 85]. Furthermore, a phase II trial of AAV2‑NGF in Alzheimer’s disease demonstrated that AAV2 delivery was safe and well‑tolerated through 24 months [86]. These data are discussed here as a reference for future translation, acknowledging that direct evidence in neurodegeneration remains lacking.

### Blocking peripheral immune cell infiltration: intervention targeting adhesion molecules and chemokine signals

While promoting lymphatic repair, directly blocking the infiltration of already activated peripheral immune cells into the CNS constitutes another therapeutic strategy. The rationale behind this strategy is that even if lymphatic function is partially restored, if pre-existing activated T cells in the periphery continue to enter the brain, the vicious cycle of neuroinflammation may persist. Therefore, simultaneously intervening in the CNS migration process of T cells may achieve synergistic effects.

Integrin α4 is a key molecule on the T cell surface mediating interaction with the blood–brain barrier (BBB). The binding of α4β1 integrin on T cells to VCAM-1 upregulated on brain microvascular endothelial cells is the initiating step for T cell adhesion, rolling, and transendothelial migration [87]. Natalizumab, a humanized monoclonal antibody against α4 integrin, has been proven effective in blocking T cell infiltration into the CNS in multiple sclerosis treatment [88, 89]. In Alzheimer's disease mouse models, Natalizumab treatment significantly reduced the number of CD4⁺ and CD8⁺ T cells in the brain by approximately 70—80%, lowered the local IFN-γ level, thereby relieving the pro-inflammatory stimulation on microglia, transforming them into M2 protective phenotypes, and improving cognitive function [90]. Notably, Natalizumab treatment does not affect the overall number of T cells in peripheral lymphoid organs but selectively blocks their migration to the CNS, thus having a relatively limited impact on systemic immune function. However, long-term application requires vigilance regarding the risk of progressive multifocal leukoencephalopathy, a serious complication caused by JC virus reactivation that limits its widespread use in non-life-threatening diseases [88]. Addressing this safety concern, researchers are developing next-generation integrin inhibitors, such as antibodies targeting the α4β1 dimer rather than individual subunits, and oral small molecule integrin antagonists, hoping to maintain efficacy while reducing immunosuppressive risks [91]. Notably, oral small-molecule integrin antagonists have already entered clinical evaluation in related neurological indications. Firategrast, an oral integrin antagonist developed by GlaxoSmithKline, demonstrated efficacy and acceptable safety in a phase II clinical trial for relapsing–remitting multiple sclerosis, providing proof-of-concept that small-molecule blockade of integrin-mediated immune cell trafficking is feasible in CNS disorders [92]. However, its application in neurodegenerative diseases such as Alzheimer's or Parkinson's disease has not yet been reported.

T cell migration to the CNS depends on the interaction between chemokine receptors on their surface and corresponding ligands within the brain. Research has found that CXCR3 and CCR5 play a core role in mediating the migration of T cells to AD brain tissue [93]. CXCR3 is involved in the formation of Aβ plaques and cognitive impairment through its ligand CXCL10. CCR5 is mainly responsible for the migration process of T cells across the BBB, thereby linking peripheral immune cells with the inflammatory response of the CNS. Astrocytes are the main source of CXCL10 in the brain of AD. Elevated CXCL10 participates in the recruitment and activation of microglia by binding to CXCR3 in the microenvironment, thereby exacerbating neuroinflammation and Aβ pathology. Microglia are the main expressers of the CCL5 receptor. They receive signals from CCL5 through CCR5, which is crucial for their chemotactic movement towards Aβ deposition sites and may further amplify inflammatory signals by generating other CCR5 ligands. In the brain of AD mouse models, CD8 + T cells highly expressed CXCR3, and CXCL10 was significantly elevated, suggesting that the CXCL10-CXCR3 axis may promote the infiltration of CD8-positive T cells into the brain, exacerbating neuroinflammation and degeneration [94]. The CCR5 antagonist Maraviroc, an already approved drug for HIV treatment with a good safety profile, is under clinical investigation for its application in Alzheimer's disease [95]. Furthermore, FTY720, as an S1P receptor modulator, induces internalization and degradation of the S1P1 receptor on lymphocyte surfaces, preventing their egress from lymph nodes, thereby reducing the number of lymphocytes entering the circulation and CNS. Preclinical studies show that FTY720 has protective effects in models of Alzheimer's disease, Huntington's disease, and amyotrophic lateral sclerosis, and its widespread use in multiple sclerosis provides a safety foundation for its translation in neurodegenerative diseases [96–100].

Clinical translation perspective: Blocking T cell infiltration into the CNS represents an attractive strategy to interrupt neuroinflammation. Natalizumab's efficacy in multiple sclerosis supports its potential in neurodegenerative diseases, but the risk of progressive multifocal leukoencephalopathy (PML) is a major concern, particularly in elderly patients who may have underlying immunosenescence. Next-generation integrin inhibitors with improved safety profiles, such as oral α4β7-selective antagonists or antibodies targeting the α4β1 dimer, are in development and may reduce PML risk. CCR5 antagonists like maraviroc, already approved for HIV, offer a repurposing opportunity with a favorable safety record, though their CNS penetrance and efficacy in neurodegeneration require validation. FTY720's broad immunosuppressive effects necessitate careful monitoring for infections and bradycardia. Stratification biomarkers such as peripheral T cell activation markers (CD69, HLA-DR) or chemokine receptor expression could help select patients with ongoing peripheral immune activation. However, it is important to note that clinical trials of anti‑CD4 monoclonal antibodies in Alzheimer's disease failed to show efficacy, and fingolimod did not meet primary endpoints in amyotrophic lateral sclerosis [96], underscoring the complexity of immune modulation in chronic neurodegeneration.

### Restoring peripheral immune homeostasis: treg modulation and immune tolerance reconstitution

Regulating the peripheral immune system itself to re-establish immune tolerance towards CNS antigens represents a more fundamental therapeutic approach. The core of this strategy lies in correcting the imbalance between effector T cells and Tregs, shifting the immune response pendulum from a pro-inflammatory direction towards an anti-inflammatory direction.

Tregs are a core cell population maintaining peripheral immune tolerance, regulating effector T cell activation and proliferation by secreting inhibitory cytokines such as IL-10, TGF-β, and IL-35, as well as through cell contact inhibition mediated by surface molecules like CTLA-4. In various neurodegenerative disease models, a decrease in the number and function of peripheral Tregs has been observed. Based on this finding, expanding Tregs becomes an intuitive strategy to restore immune homeostasis [101–104].

IL-2 is a key cytokine for the survival and proliferation of Tregs, but high-dose IL-2 also activates effector T cells and NK cells. Researchers have found that when IL-2 forms complexes with specific anti-IL-2 antibodies that bind to a particular epitope, it can selectively expand Tregs that highly express IL-2Rα, with minimal effect on effector T cells. In Alzheimer's disease mice, intraperitoneal injection of low-dose recombinant human IL-2 increases the proportion of Tregs in the spleen and lymph nodes by threefold, while enhancing Foxp3 expression and suppressive function [105]. Clinical studies have shown that in AD patients, a 5-day low-dose IL-2 course every 4 weeks is safe and can effectively and continuously amplify Tregs [106]. This treatment strategy has demonstrated the potential to regulate peripheral inflammation and improve the core biomarker of AD (Aβ42) in cerebrospinal fluid, and has provided preliminary evidence for slowing down cognitive decline [106]. The adoption transfer of Tregs also shows a protective effect. Tregs were isolated from the spleens of healthy mice, expanded in vitro and intravenously injected into mice with Alzheimer's disease. This reduced the deposition of Aβ plaques in the cerebral cortex and hippocampus, and decreased the activation of microglia and the levels of pro-inflammatory cytokines (IL-6, IFN-γ, IL-17A) in the spleen. Meanwhile, the anti-inflammatory factor IL-10 was increased, significantly improving cognitive function [107].

Antigen-specific immune tolerance induction is a more precise immunomodulatory strategy. Administering CNS antigens via nasal, oral, or subcutaneous routes can induce antigen-specific Tregs or regulatory B cells, thereby re-establishing tolerance to specific antigens without causing widespread immunosuppression [108]. The nasal mucosa, rich in dendritic cells and possessing a microenvironment conducive to tolerance induction, is an advantageous route for delivering CNS antigens. In Alzheimer's disease mice, intranasal administration of Aβ1—42 peptide induced Th2/Th3 type anti-inflammatory immune responses characterized by IgG1/IgG2b antibodies and IL-4/IL-10/TGF-β expression in the brain, significantly reducing the hippocampal Aβ plaque burden by 60% [109]. Research has found that mice administered α -synuclein aggregates via the nose not only exhibited Parkinson's disease-like behavioral deficits and dopaminergic neurochemical changes, but also triggered humoral immune protective responses against α -synuclein and/or dopamine [110]. Although the original intention of this study was to build a model, the immune activation phenomenon it observed provided early and important experimental evidence for exploring whether nasal administration might induce protective immunity. The mechanisms of oral tolerance involve the special microenvironment of gut-associated lymphoid tissue; the liver also plays an important role in the processing and presentation of orally administered antigens, but its application research in CNS neurodegenerative diseases is currently relatively limited. In vitro modification and reinfusion of dendritic cells is another precise strategy: dendritic cells are treated in vitro with anti-inflammatory factors or transfected to express regulatory molecules, then loaded with CNS antigens and reinfused into the body, where they can induce the expansion of antigen-specific Tregs in vivo. This strategy has shown potential in autoimmune disease models, and its exploration in neurodegenerative diseases is ongoing.

Clinical translation perspective: Restoring immune tolerance through Treg expansion or antigen‑specific approaches offers a more fundamental and durable solution. Low‑dose IL‑2 therapy has demonstrated safety and Treg expansion in early Alzheimer's trials [106]; NCT05870800), but the optimal dose, duration, and combination with other agents remain to be defined. Antigen‑specific tolerance induction via intranasal or oral administration carries minimal systemic immunosuppression risk but requires careful antigen selection and dosing to avoid inadvertent immune activation. Adoptive Treg transfer is logistically challenging and may be reserved for severe or rapidly progressive cases. Biomarkers such as peripheral Treg frequency (Foxp3⁺), antigen‑specific T cell clones (tetramer analysis), and cytokine profiles will be essential for monitoring treatment response and guiding personalized regimens.

Proposed modified strategies for restoring immune tolerance. Based on the limitations of current approaches, we propose the following refined strategies: (1) Low‑dose IL‑2 plus intranasal antigen co‑administration – sequential rather than concurrent delivery may reduce the risk of activating effector T cells; a staggered protocol (IL‑2 priming for one week followed by intranasal Aβ or α‑synuclein) could promote antigen‑specific Treg expansion while minimizing bystander inflammation. This approach builds on established evidence that low‑dose IL‑2 safely expands Tregs in AD patients [106], while intranasal antigen delivery effectively induces immune tolerance [109]. (2) Engineered Tregs with CNS‑homing receptors – adoptive transfer of Tregs transduced with CX3CR1, CXCR3, or α4β7 to improve trafficking to deep cervical lymph nodes and inflamed brain regions. This concept is supported by studies showing that CX3CR1‑transduced Tregs home to the forebrain and reduce neuroinflammation in AD mouse models [111], and that TCR‑engineered Aβ‑specific Tregs exhibit brain homing and reduce amyloid pathology [72]. (3) Nanoparticle‑encapsulated tolerogenic cocktails – co‑delivery of IL‑2, TGF‑β, and CNS antigens via brain‑targeted nanoparticles (e.g., RVG‑modified liposomes) to directly condition dendritic cells in the draining lymph nodes. RVG‑functionalized nanoparticles have been successfully used for brain‑targeted delivery in AD models [112, 113], providing a platform for this strategy. (4) Biomarker‑guided intermittent dosing – using peripheral Treg frequency (Foxp3⁺) and serum IL‑10 levels as pharmacodynamic markers to individualize IL‑2 administration (e.g., only when Treg/CD4⁺ ratio falls below 5%). In clinical trials, Foxp3 expression has been used as a pharmacodynamic marker to monitor Treg expansion and guide IL‑2 dosing [106]. These strategies are currently being evaluated in preclinical models and warrant clinical exploration.

### Combination treatment strategies: exploring synergistic effects

**Fig. 3:**
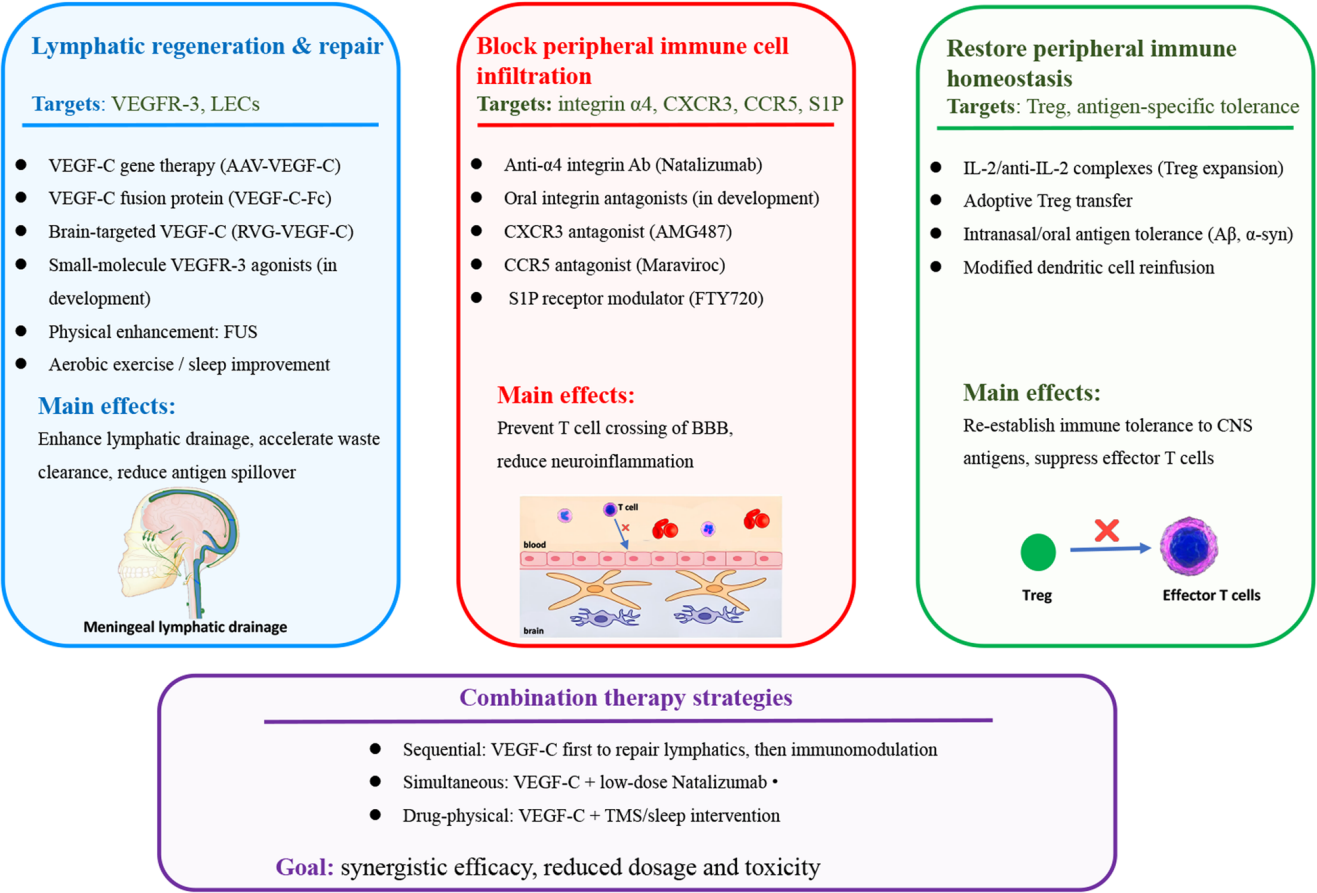
Therapeutic strategies targeting the meningeal lymphatic vessel-peripheral immune axis. Three major therapeutic approaches are depicted with their corresponding targets, representative interventions, and main effects. Left: Lymphatic regeneration and repair targets VEGFR-3 on lymphatic endothelial cells through strategies including VEGF-C gene therapy, VEGF-C fusion proteins, focused ultrasound, and non-pharmacological interventions. Middle: Blocking peripheral immune cell infiltration targets adhesion molecules and chemokine signals (integrin α4, CXCR3, CCR5, S1P receptors) using Natalizumab, Maraviroc, FTY720, and emerging oral antagonists. Right: Restoring peripheral immune homeostasis targets regulatory T cells and antigen-specific tolerance through IL-2 complexes, Treg transfer, and intranasal/oral antigen administration. Bottom: Combination strategies integrate these approaches sequentially or simultaneously to achieve synergistic efficacy while minimizing individual drug toxicities

Figure 3 summarizes these therapeutic approaches targeting different components of the meningeal lymphatic vessel-peripheral immune axis. The core of this strategy lies in the dynamic intervention of the disease process. Based on an in-depth understanding of the neuroimmune cascade reaction, we propose a sequential combination protocol: Firstly, initiate VEGF-C-based lymphatic regeneration and repair in the early treatment stage. This step aims to accelerate the clearance of risk-related molecular patterns in the CNS, such as Aβ, and reduce their spillover to peripheral lymphoid organs. Subsequently, on the basis of a reduced CNS antigen load, Treg modulators or tolerance inducers are administered, aiming to re-establish immune tolerance to CNS antigens and block the initiated autoimmune response.

The logic of this strategy is supported by previous research. [67] found in a mouse model of Alzheimer's disease that enhanced lymphatic drainage mediated by VEGF-C significantly improved the clearance effect of anti-Aβ antibodies, suggesting that improving lymphatic function may be a prerequisite for the success of subsequent immune intervention [67]. The mechanistic rationale for sequential VEGF-C followed by immunomodulation is twofold: First, initial lymphatic repair reduces the burden of CNS-derived antigens reaching peripheral lymphoid organs, thereby decreasing the antigenic drive that sustains autoreactive T cell activation. Second, by lowering the concentration of abnormally modified antigens in the deep cervical lymph nodes, VEGF-C treatment creates a more favorable microenvironment for tolerance induction, allowing immunomodulatory agents such as low-dose IL-2 to focus on maintaining regulatory T cell function rather than contending with a high-load pro-inflammatory milieu. This study also found that the combination of VEGF-C treatment and anti-Aβ antibodies demonstrated a synergistic effect in clearing Aβ plaques in multiple AD mouse models (5xFAD, APPswe, J20), and significantly reduced the proliferation and activation of microglia around Aβ plaques [67]. Therefore, the combination of enhanced lymphatic drainage through VEGF-C and immunotherapy is a promising therapeutic strategy.

Beyond drug combinations, integrating pharmacological with non-pharmacological interventions is also noteworthy. Combining VEGF-C treatment with transcranial magnetic stimulation or transcranial direct current stimulation may synergistically promote lymphatic function by enhancing neuroplasticity and cerebral blood flow [114, 115]. Non-pharmacological interventions like improving sleep hygiene and optimizing sleep architecture, combined with immunomodulatory drugs, hold promise for intervening in disease progression across multiple dimensions simultaneously. As understanding of the meningeal lymphatic vessel-peripheral immune axis deepens, more combination treatment strategies will be developed and enter preclinical and clinical evaluation.

## Perspectives and challenges

### Key issues in clinical translation

Translating therapies targeting the meningeal lymphatic vessel–peripheral immune axis from the laboratory to the clinic still faces numerous challenges, with in vivo assessment of human meningeal lymphatic vessels being the foremost issue [116]. Currently, functional assessment primarily relies on dynamic contrast-enhanced MRI (DCE-MRI), which has revealed meningeal lymphatic dysfunction in patients with cerebral small vessel disease [117], episodic migraine [118], and cognitive impairment [119]. However, this method has substantial limitations, including limited spatial resolution and lack of standardization [116]. Developing specific tracers is a key direction for improving assessment accuracy. Novel VEGFR-3-targeted PET tracers are under active development, and a recent review comprehensively summarized advances in VEGFR-targeted molecular imaging [120]. Additionally, the DTI-ALPS index has been evaluated as a surrogate marker of glymphatic clearance in large population-based studies, showing correlations with age, vascular risk, and cognitive scores [121]. A systematic review and meta-analysis further confirmed that DTI-ALPS is significantly reduced across the Alzheimer’s disease continuum and correlates with cognitive decline [122]. Emerging clinical studies are beginning to apply these techniques in patient cohorts, with early reports suggesting that lymphatic dysfunction correlates with cognitive decline and may serve as a predictive biomarker for disease progression [116].

The timing of intervention is another critical issue in clinical translation. The preclinical phase of neurodegenerative diseases lasts for decades. During this prolonged period, questions regarding when meningeal lymphatic dysfunction initiates, when it progresses to an irreversible stage, and when it becomes an ideal window for therapeutic intervention currently lack clear answers. Establishing large longitudinal cohorts, combining lymphatic function imaging, cerebrospinal fluid biomarker detection, and cognitive assessment, can help define the key time point of “lymphatic failure phase” and its temporal relationship with cognitive decline. Within this framework, future clinical trials may need to initiate intervention at very early or even preclinical stages of the disease, posing higher demands on biomarker screening and subject recruitment.

Safety considerations require particularly thorough evaluation before any therapeutic strategy advances to clinical application [84, 123]. These can be categorized into short‑term and long‑term concerns. Short‑term safety concerns include acute inflammatory responses to viral vectors (for gene therapy), infusion‑related reactions (for biologics), and potential off‑target effects on peripheral lymphatic tissues [84]. Phase I trials should incorporate intensive monitoring during the first week post‑treatment. Long‑term safety concerns are more complex. First, regarding pro‑lymphangiogenic therapies: prolonged VEGF‑C stimulation raises theoretical risks of promoting tumor growth or metastasis in individuals with occult malignancies, as the VEGF‑C/VEGFR‑3 axis promotes lymphangiogenesis and metastasis in a range of human tumors [124], necessitating baseline and periodic cancer screening. Second, regarding immunomodulatory interventions: excessive peripheral immunosuppression could increase susceptibility to infections and impair tumor immune surveillance. The risk of progressive multifocal leukoencephalopathy (PML) associated with integrin inhibitors like natalizumab is well‑documented, with risk stratification based on JC virus antibody index [125, 126], and even next‑generation agents may carry some residual risk. Monitoring of JC virus DNA in cerebrospinal fluid and peripheral blood, as well as CD4⁺ T cell counts, should be considered [125]. The ethical implications of long‑term immunosuppression in non‑life‑threatening diseases must also be carefully weighed, particularly in early‑stage or preclinical individuals who may derive uncertain benefit.

Despite the promise of these strategies, it is important to acknowledge the historical challenges in translating neuroimmunology therapies. For example, anti‑CD4 monoclonal antibodies failed to show efficacy in Alzheimer's disease trials, and fingolimod, while beneficial in multiple sclerosis, did not meet primary endpoints in amyotrophic lateral sclerosis [96]. These failures highlight the complexity of immune modulation in chronic neurodegenerative conditions and the need for careful patient selection and trial design. Furthermore, many biologics and small molecules face hurdles in crossing the BBB, limiting their access to CNS targets. In neurodegenerative diseases, where pathology is widespread within the parenchyma, achieving sufficient CNS concentrations of immunomodulatory agents may be critical. Novel delivery approaches, such as focused ultrasound‑mediated BBB disruption, intranasal administration, or conjugation to BBB‑shuttling molecules (e.g., transferrin receptor antibodies), are being explored to overcome this challenge but require further validation in preclinical models and clinical trials.

Resolution of these safety concerns need to be gradually clarified through well-designed preclinical studies across multiple species with extended observation periods, followed by phase I clinical trials incorporating comprehensive safety monitoring systems. Importantly, recent advances in lymphatic imaging and immune profiling are now enabling the design of early-phase clinical trials that can assess both efficacy and safety in parallel.

### Future research directions

The application of single‑cell and spatial transcriptomic technologies will reveal new mechanisms at the cellular level. Future research needs to resolve, at single‑cell resolution, the dynamic transcriptional changes of meningeal lymphatic endothelial cells, lymph node stromal cells, and various immune cell subsets during disease progression. Spatial transcriptomics will further map these molecular changes to anatomical locations, constructing a cellular atlas of disease‑related lymphatic remodeling. This resource will not only aid in understanding disease mechanisms but also discover new intervention targets; for example, molecules expressed on the surface of specific endothelial cell subsets could become new targets for drug delivery or imaging tracers.

Patient heterogeneity in lymphatic function and immune status underscores the need for biomarker‑guided stratification. As summarized in Table 1, lymphatic drainage efficiency measured by dynamic contrast‑enhanced MRI (DCE‑MRI) and CSF levels of Aβ and p‑tau could identify individuals with predominant lymphatic impairment who may benefit from pro‑lymphangiogenic therapies. Conversely, peripheral immune phenotyping—including Treg frequency, expression of activation markers (CD69, HLA‑DR) on T cells, and detection of antigen‑specific T cell clones via MHC tetramers—may guide the use of immunomodulatory agents. Genetic factors such as APOE genotype could also influence meningeal lymphatic function; although direct evidence is limited, APOE4 is known to compromise blood–brain barrier integrity and may similarly affect lymphatic endothelial cells, warranting further investigation. Integrating these multimodal biomarkers into clinical trial designs will enable enrichment of homogeneous patient populations and facilitate personalized therapeutic interventions.

**Table 1:** Therapeutic strategies targeting the meningeal lymphatic-peripheral immune axis: status, challenges, and biomarkers

Strategy	Representative Interventions	Developmental Stage	Key Challenges for Translation	Potential Stratification Biomarkers	Safety Concerns
Lymphatic regeneration/repair	AAV-VEGF-C, VEGF-C-Fc, RVG-VEGF-C, focused ultrasound (FUS)	Preclinical (AAV-VEGF-C in mouse models); FUS in early clinical trials (phase I for other indications)	Long-term safety, tumor risk (theoretical due to VEGFR-2 cross-reactivity), delivery efficiency, monitoring of lymphatic function	Lymphatic drainage efficiency (DCE-MRI), CSF Aβ/p-tau levels	Potential for promoting tumor growth/angiogenesis; off-target effects on peripheral lymphatics
Blocking immune infiltration	Natalizumab (anti-α4), Maraviroc (CCR5 antagonist), FTY720 (fingolimod)	Natalizumab approved for MS (phase IV); Maraviroc approved for HIV (phase II for AD ongoing, NCT05100719); FTY720 approved for MS (phase II for ALS completed, NCT03235791)	PML risk (Natalizumab), infection susceptibility, need for CNS-penetrant agents (Maraviroc), bradycardia/immunosuppression (FTY720)	Peripheral T cell activation markers (CD69, HLA-DR), CCR5/CXCR3 expression	PML (JC virus reactivation); increased risk of infections; cardiovascular effects (FTY720)
Restoring immune tolerance	IL-2/anti-IL-2 complexes, intranasal antigens (Aβ, α-syn), Treg transfer	Preclinical (IL-2 complexes in AD mice); Phase II for low-dose IL-2 in AD completed (NCT05870800)	Antigen specificity, durability of tolerance, risk of generalized immunosuppression, manufacturing complexity (Treg transfer)	Treg frequency (Foxp3⁺), antigen-specific T cell clones (tetramer), cytokine profiles	Potential for systemic immunosuppression; autoimmune risk; cytokine release syndrome
Combination strategies	Sequential (VEGF-C → Treg therapy); simultaneous (VEGF-C + low-dose Natalizumab)	Preclinical	Optimal sequencing, dosing, potential drug-drug interactions, overlapping toxicity, regulatory complexity	Multi-modal: lymphatic function + immune phenotype	Accumulation of individual toxicities; unpredictable interactions

The functional coupling between meningeal lymphatic vessels and the BBB is a new direction worthy of in-depth exploration. Preliminary evidence suggests these two interfaces do not operate independently but have complex interactions: meningeal lymphatic drainage obstruction can lead to increased intracranial pressure, subsequently affecting BBB tight junctions; infiltration of peripheral immune cells through the damaged BBB can release cytokines, further damaging lymphatic endothelial cells. Understanding this synergistic regulatory mechanism may yield new therapeutic ideas for simultaneously improving both interfaces. For example, targeting molecules co-expressed by both interfaces, such as VEGF-B or ANGPT2, could produce broader neurovascular unit protective effects.

Personalized treatment strategies are a natural extension of precision medicine in this field. Significant heterogeneity exists among patients in terms of meningeal lymphatic function status, peripheral immune phenotype, genetic background, and disease stage, implying that a single treatment strategy may not benefit all patients. Based on lymphatic function imaging results, patients could be stratified into “drainage impairment predominant” and “relatively preserved drainage function” types; the former might benefit more from pro-lymphangiogenic therapies like VEGF-C, while the latter might require immunomodulatory interventions. Based on peripheral immune phenotype, such as the effector T cell/Treg ratio or the expansion degree of specific T cell clones, the selection and dosage adjustment of immunomodulatory drugs could be guided. Genetic factors like APOE genotype and TREM2 variants may also influence patient responses to specific treatments. The design of future clinical trials needs to fully account for these heterogeneity factors, using biomarker-guided enrichment strategies to validate treatment efficacy in more homogeneous patient populations. Early-phase clinical studies are now beginning to incorporate these stratification approaches, paving the way for precision immunomodulation in neurodegenerative diseases.

## Conclusion

The discovery of meningeal lymphatic vessels has provided a revolutionary new framework for understanding the pathogenesis of neurodegenerative diseases. The proposal of the meningeal lymphatic vessel-peripheral immune axis integrates central protein aggregation, local neuroinflammation, and peripheral immune responses into a unified pathological process, revealing the intrinsic connections between these seemingly independent pathological features. Within this framework, classic neurodegenerative diseases like Alzheimer’s disease and Parkinson’s disease are no longer viewed solely as intrinsic neuronal disorders but as complex diseases involving the entire body system. Therapeutic strategies targeting this axis—from VEGF-C-mediated lymphatic regeneration and repair, through integrin blockade inhibiting T cell infiltration, to regulatory T cell expansion for immune tolerance reconstitution—have shown encouraging efficacy in preclinical studies. As neurologists, we are witnessing a profound shift in the therapeutic paradigm for CNS diseases from a “neurocentric” view towards “integrated neuro-immune-vascular network regulation”. This shift holds promise for breakthrough advancements in diseases like Alzheimer’s and Parkinson’s, which currently lack effective treatments. In future clinical practice, we may be able to intervene effectively in the progression of neurodegenerative diseases by “opening the brain’s drainage valve” while simultaneously modulating the aggressiveness of the peripheral immune system. Although numerous challenges lie ahead, this emerging field has already opened a promising new direction for the treatment of neurodegenerative diseases.

## Data Availability

No datasets were generated or analysed during the current study.
